# First Trimester Screening for Preterm Preeclampsia in the United Kingdom: A Cost‐Effectiveness Analysis

**DOI:** 10.1111/1471-0528.70294

**Published:** 2026-07-01

**Authors:** Mohammad A. Ani, Liona C. Poon, Laura A. Magee, A. Joy Allen, Kypros H. Nicolaides

**Affiliations:** ^1^ Roche Diagnostics UK and Ireland Burgess Hill UK; ^2^ Department of Obstetrics and Gynaecology The Chinese University of Hong Kong Shatin Hong Kong Special Administrative Region of China; ^3^ Harris Birthright Research Centre for Fetal Medicine, King's College Hospital London UK; ^4^ Department of Women and Children's Health School of Life Course and Population Sciences, King's College London London UK

**Keywords:** algorithm, aspirin, cost‐effectiveness, first‐trimester, prediction, preeclampsia, prevention, screening

## Abstract

**Objective:**

To evaluate the cost‐effectiveness of the Fetal Medicine Foundation (FMF) strategy, compared with the National Institute for Health and Care Excellence (NICE) strategy, for first‐trimester screening for preterm preeclampsia (PE) in the United Kingdom (UK).

**Design:**

Cost‐effectiveness analysis.

**Setting:**

UK National Health Service and personal social services perspective.

**Population:**

A total of 10 000 simulated patients with singleton pregnancies at 11–13 weeks' gestation, across a lifetime time horizon.

**Methods:**

A decision‐tree model was developed to perform a cost‐effectiveness analysis. In the base‐case analysis, NICE‐recommended screening was compared with FMF screening, using maternal factors, mean arterial pressure (MAP), uterine artery pulsatility index (UtA‐PI) and placental growth factor (PlGF). The model assumed that patients identified as high‐risk for PE were prescribed 150 mg aspirin daily until 36 weeks' gestation. Scenario analyses varied PE incidence, aspirin adherence and biomarker combinations of FMF strategy components.

**Main Outcome Measure:**

Incremental cost‐effectiveness ratios (ICERs) were calculated using incremental costs and quality‐adjusted life years (QALYs). Dominant ICERs demonstrated lower costs and higher QALYs.

**Results:**

Compared with NICE‐recommended screening, the FMF strategy demonstrated a cost‐saving of £3191 and QALY gain of 0.92 per 10 000 patients (dominant ICER), with a cost‐saving of £199 per preterm PE case avoided. In scenario analyses, the FMF strategy was cost‐effective across 3%, 5% and 7% PE incidence, and 75% and 100% aspirin adherence. The base‐case FMF strategy (maternal factors + MAP + UtA‐PI + PlGF) was the most clinically effective option.

**Conclusions:**

The FMF strategy was more cost‐effective versus the NICE strategy for first‐trimester preterm PE screening in the UK.

## 1 Introduction

Preeclampsia (PE) is a global problem, which affects ~2%–5% of pregnancies, and is responsible for ~46 000 maternal deaths and 500 000 foetal/newborn deaths annually [1]. PE prevention is key, as clinical presentation is highly variable, severe disease may be asymptomatic and care is complex [2]. Identification of preterm PE risk is undertaken in the first‐trimester of pregnancy, when preventive therapy is initiated with low‐dose aspirin [3, 4], and enhanced monitoring is offered throughout pregnancy, to minimise maternal and perinatal morbidity and mortality [5].

PE risk assessment can be based on clinical risk factors, as developed by the United Kingdom (UK) National Institute for Health and Care Excellence (NICE) [5], or multivariable modelling using the competing risks model developed by the Fetal Medicine Foundation (FMF) [6, 7]. Under the NICE guidelines, patients are considered at high risk of developing PE if they have one major risk factor (hypertensive disease in previous pregnancy, chronic kidney disease, autoimmune disease, diabetes mellitus or chronic hypertension), or ≥ 2 moderate risk factors (nulliparity, age ≥ 40 years, interpregnancy interval > 10 years, body mass index at first visit ≥ 35 kg/m^2^, family history of PE or multifoetal pregnancy) [5]. In contrast, the FMF multivariable logistic model uses Bayes' theorem to combine the a priori risk of PE from maternal factors, with various combinations of uterine artery pulsatility index (UtA‐PI), mean arterial pressure (MAP) and placental growth factor (PlGF), using ‘multiple of the median’ values (which adjust for gestational age and maternal factors that provide substantive contribution to their value) [6, 7, 8]. In the multicentre, prospective screening programme for PE (SPREE) study, the detection rate for preterm PE was 82.4% with the FMF strategy (maternal factors + MAP + UtA‐PI + PlGF), compared with 40.8% using NICE‐recommended screening [9].

As PE costs the National Health Service (NHS) ≈£300 million annually, there remains an unmet need for model‐based cost‐effectiveness analyses of first‐trimester screening methods for PE in the UK [10, 11]. Studies have demonstrated the cost‐effectiveness of various biomarker combinations of the FMF strategy in Canada, Belgium, Germany and Switzerland [12, 13, 14]. In a real‐world, retrospective analysis in a London hospital (*n* = 5957), the FMF strategy using maternal factors + MAP + pregnancy‐associated plasma protein‐A (PAPP‐A) was associated with a cost‐saving of £9.06 per pregnancy screened, compared with NICE‐recommended screening [15].

This study is the first model‐based economic analysis of first‐trimester screening for PE in the UK. We evaluated the cost‐effectiveness of the FMF strategy (maternal factors + MAP + UtA‐PI + PlGF) versus NICE‐recommended screening.

## 2 Methods

### 2.1 Model Structure

A decision‐tree model was developed to perform a cost‐effectiveness analysis of first‐trimester screening for preterm PE risk, from the UK NHS and personal social services (PSS) perspective. The FMF strategy was compared with the NICE guideline standard‐of‐care (SoC) strategy. A total of 10 000 patients with singleton pregnancies at 11–13 weeks' gestation were simulated across a lifetime time horizon, consistent with the NICE reference case for economic evaluations [16]. Lifetime QALYs were included to capture the long‐term health consequences associated with maternal and neonatal outcomes. Cost differences between strategies were modelled only during the 1‐year perinatal period, as the association between PE and long‐term health problems may not be causal. Long‐term costs were assumed to be equal between the two strategies. The model was built in both R‐studio and Microsoft Excel for validation purposes. For both FMF and NICE strategies, the clinical pathway of patients undergoing first‐trimester screening is presented in Figure 1A; decision tree model structures for each strategy are reported in Figure 1B. Each health state was associated with costs and health‐related quality of life weights. In the base‐case analysis, FMF screening using maternal factors + MAP + UtA‐PI + PlGF was compared with NICE‐recommended screening.

**FIGURE 1:**
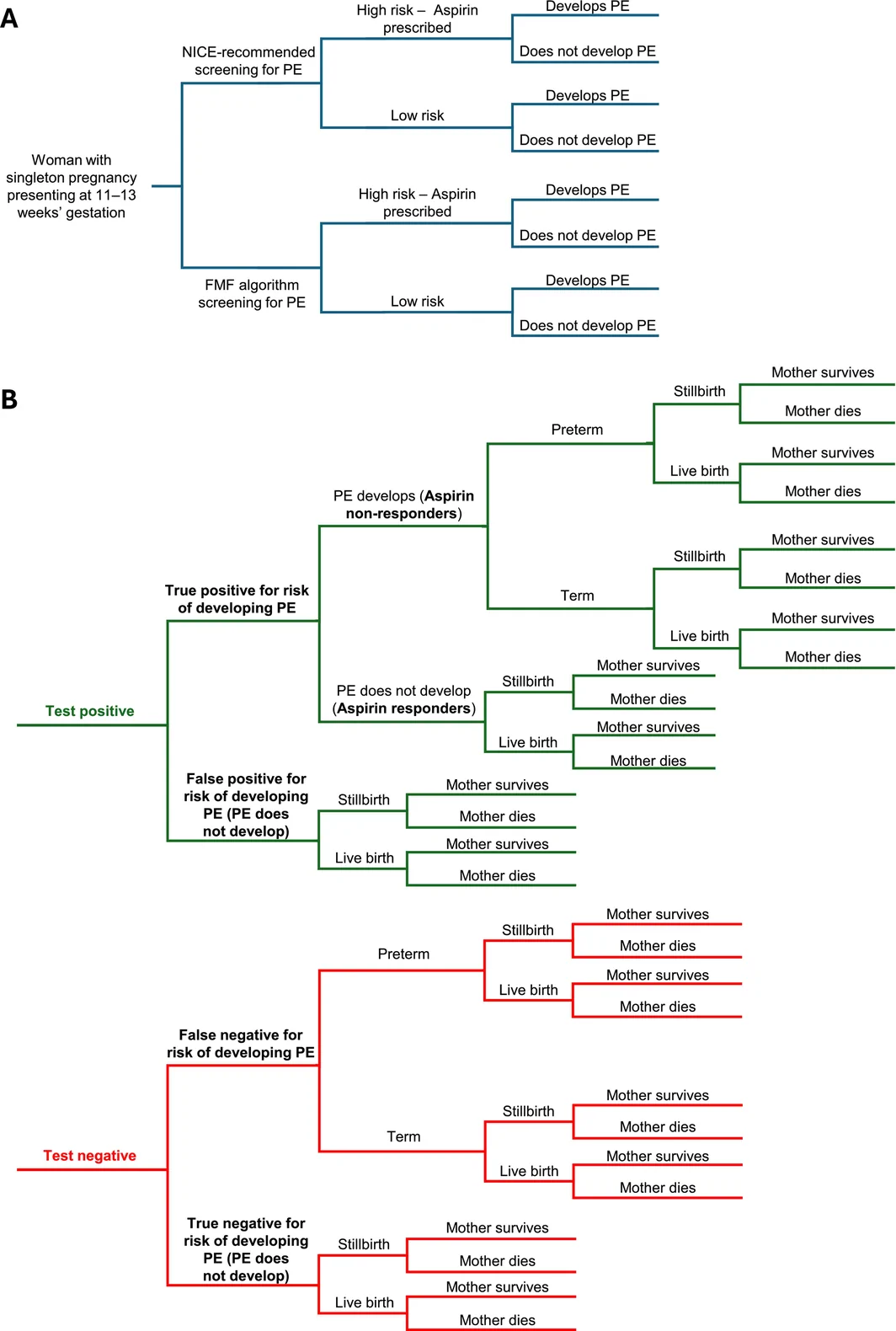
(A) The clinical pathway of women undergoing first‐trimester screening for both the NICE and FMF strategies. (B) Decision tree model structure for the NICE strategy and FMF strategy. FMF, Fetal Medicine Foundation; NICE, National Institute for Health and Care Excellence; PE, preeclampsia.

### 2.2 Key Assumptions/Parameters

Data were obtained from published literature, national guidelines, public cost collections databases and expert opinions. Per expert opinion, the cost of MAP was assumed to be £0 in both arms, as blood pressure measurement is SoC across visits. Each patient was assumed to have had two midwifery contacts: one initial appointment at 11–13 weeks' gestation to discuss PE risk and undergo counselling for measures to prevent PE, and one follow‐up appointment to assess pregnancy progress. The model assumed that patients identified as high‐risk for PE were prescribed 150 mg aspirin daily, from 11 to 13 weeks' until 36 weeks' gestation, whereas patients identified as low‐risk were not prescribed aspirin. Patients could either develop preterm PE, term PE or not develop PE; those who developed PE were admitted to hospital prior to birth, treated with labetalol (for hypertension) and corticosteroids (patients who developed preterm PE only), and underwent relevant assessment (with maternal blood tests, including kidney function tests for end‐organ dysfunction, and foetal assessment, as per NICE guidelines) [17, 18]. Management after diagnosis was identical across both arms and risk categories. Vaginal birth and emergency or elective caesarean delivery modes were accommodated in the model, as well as outcomes such as stillbirth or live birth, along with the possibility of maternal death (not associated with additional cost). The model assumed that babies could be admitted to the neonatal intensive care unit (NICU) or special care baby unit (SCBU), as appropriate. Patients were assigned a disutility for: aspirin therapy to account for possible side effects, each type of birth, stillbirth and NICU admission for their child.

Strategy performance, epidemiology and aspirin input parameters for the base‐case analysis are reported in Table 1; overall PE incidence of 3% and aspirin adherence of 75% were used in both arms, based on expert opinion. Input parameters for probability and cost of each event are reported in Tables S1 and S2. Utilities and disutilities associated with short‐ and long‐term effects on quality of life outcomes for the patient, and short‐term effects for the baby due to NICU admission, were extracted from NICE Diagnostic Guidance (HTG630, previously DG49) and published literature (Table S3) [15, 17]. Parameters for length of hospital stay in the NICU and SCBU for preterm PE, term PE and no PE are reported in Table S4.

**TABLE 1:** Key input parameters for the base‐case analysis.

Parameter	Estimate	Distribution	Alpha	Beta	95% CI	References
Sensitivity preterm PE						
NICE	41.5%	Beta	59	83	0.34–0.50	Tan et al. [9]^a^
FMF (maternal factors + MAP + UtA‐PI + PlGF)	81.7%	Beta	116	26	0.75–0.88	Tan et al. [9]^a^
Specificity preterm PE						
NICE	90.1%	Beta	14 658	1616	0.90–0.91	Tan et al. [9]^a^
FMF (maternal factors + MAP + UtA‐PI + PlGF)	90.4%	Beta	14 715	1559	0.90–0.91	Tan et al. [9]^a^
Sensitivity term PE						
NICE	30.2%	Beta	100	231	0.25–0.35	Tan et al. [9]^a^
FMF (maternal factors + MAP + UtA‐PI + PlGF)	42.6%	Beta	141	190	0.37–0.48	Tan et al. [9]^a^
Specificity term PE						
NICE	90.3%	Beta	14 699	1575	0.90–0.91	Tan et al. [9]^a^
FMF (maternal factors + MAP + UtA‐PI + PlGF)	90.6%	Beta	14 740	1534	0.90–0.91	Tan et al. [9]^a^
Epidemiology						
Incidence	3.0%	Beta	97	3136	0.024–0.036	Expert opinion based on previous literature [19, 20]
Proportion of preterm PE	28.6%	Beta	180	450	0.25–0.32	Rolnik et al. [4]^a^
Proportion of term PE	71.4%	—	—	—	0.68–0.75	Rolnik et al. [4]^a^, ^b^
Aspirin						
Treatment effect (odds ratio)	0.38	Beta	62	101	0.31–0.46	Rolnik et al. [4]
Adherence rate	75%	—	—	—	—	Expert opinion

Abbreviations: FMF, Fetal Medicine Foundation; MAP, mean arterial pressure; NICE, National Institute for Health and Care Excellence; PE, preeclampsia; PlGF, placental growth factor; UtA‐PI, uterine artery pulsatility index.

^a^ Estimates were calculated from cited source.

^b^ Proportions of term PE in the probabilistic sensitivity analysis were derived using 1‐ proportions for preterm PE.

### 2.3 Analyses

The primary outcomes included: total costs and quality‐adjusted life years (QALYs) for each screening strategy; incremental costs and QALYs; incremental cost‐effectiveness ratios (ICER); number of cases and cost per case of preterm PE avoided. ICERs were defined as the ratio between the change in cost to change in QALYs for the FMF strategy compared with NICE‐recommended screening. Dominant ICERs demonstrated lower costs and higher QALYs.

### 2.4 Scenario Analyses

Various scenario analyses were modelled in addition to the base‐case. Scenario analysis A considered PE incidences of 3%, 5% and 7%. Scenario analysis B varied aspirin adherence to levels of 25%, 50%, 75% and 100%. Scenario analysis C considered different biomarker combinations of the FMF strategy: maternal factors + MAP; maternal factors + MAP + UtA‐PI; maternal factors + MAP + PlGF and maternal factors + MAP + UtA‐PI + PlGF. Scenario analysis D modelled short‐term disutility parameters to directly compare costs and QALYs of each strategy across a 1‐year time horizon. The parameters varied in scenario analyses A‐D are reported in Table S5. Scenario analysis E modelled hospitalisation rates of 100% for preterm and term PE, as well as 80% and 100% rates for preterm and term PE, respectively. In addition, threshold analyses were performed to determine the threshold at which the FMF strategy becomes dominant versus the NICE strategy, and becomes cost‐effective or is no longer cost‐effective across different willingness‐to‐pay (WTP) thresholds using different hospitalisation rates (threshold analysis A) and different costs of UtA‐PI (threshold analysis B).

### 2.5 Sensitivity Analyses

To assess the impact on results of joint parameter uncertainty, a probabilistic sensitivity analysis (PSA) was performed, with a total of 10 000 simulations to build a cost‐effectiveness plane and acceptability curve across various WTP thresholds. Utility decrements were sampled as positive beta‐distributed values and then applied as negative disutilities. To explore the value of collecting more information on relevant parameters, an expected value of perfect information (EVPI) analysis was performed using PSA simulations. A deterministic one‐way sensitivity analysis was performed to assess the impact on the ICER of varying individual parameters by ±5%. A tornado plot was generated to display the change in ICERs for the parameters that had the greatest impact on the results.

## 3 Results

### 3.1 Base‐Case

Total costs for the FMF (maternal factors + MAP + UtA‐PI + PlGF) and NICE strategies per 10 000 patients screened were £47 758 351and £47 761 543, respectively (Table 2). Total QALYs were 171 859.57 for the FMF strategy and 171 858.66 for the NICE strategy. Using the FMF strategy there were 53 cases of preterm PE per 10 000 patients, and 69 with the NICE strategy; as such, adoption of FMF screening is predicted to avoid an additional 16 cases of preterm PE (23%) per 10 000 people screened (Table S6).

**TABLE 2:** Base‐case results.

Strategy	Total cost (£)	Total QALYs	Incremental costs (£)	Incremental QALYs	ICER (£/QALYs)	Cost per preterm PE case avoided
NICE	47 761 543	171 858.66	—	—	—	—
FMF (maternal factors + MAP + UtA‐PI + PlGF)	47 758 351	171 859.57	−3191	0.92	Dominant	−199

Abbreviations: FMF, Fetal Medicine Foundation; ICER, incremental cost‐effectiveness ratio; MAP, mean arterial pressure; NICE, National Institute for Health and Care Excellence; PE, preeclampsia; PlGF, placental growth factor; QALY, quality‐adjusted life year; UtA‐PI, uterine artery pulsatility index.

The FMF strategy led to a cost‐saving of £3191, with a QALY gain of 0.92 per 10 000 patients versus NICE‐recommended screening. As such, the ICER for the FMF strategy was dominant (i.e., had lower costs and higher QALYs compared with NICE‐recommended screening). Adoption of the FMF strategy was associated with a cost‐saving of £199 per preterm PE case avoided. Base‐case clinical outcomes per 10 000 patients screened, and cost‐breakdown per one patient screened, are shown in Tables S6 and S7.

### 3.2 Scenario Analysis A: Incidence of PE

PE incidence was varied at 3% (base‐case), 5% and 7%. The FMF strategy avoided more cases of preterm PE at each PE incidence: 16.0, 26.7 and 37.3 cases avoided at 3%, 5% and 7% PE incidence, respectively (Table 3). The incremental costs for the FMF (versus NICE) strategy for 3%, 5% and 7% PE incidence were: –£3191, −£196 368 and –£389 545, respectively (i.e., cost savings), with incremental QALYs of 0.92, 1.52 and 2.13, respectively. Therefore, the FMF strategy remained the dominant approach compared with NICE‐recommended screening across all PE incidence rates modelled.

**TABLE 3:** Scenario analyses for PE incidence, aspirin adherence rate and different biomarker combinations of the FMF strategy versus NICE strategy (per 10 000 patients screened).

Scenario	Strategy	Total costs (£)	Total QALYs	Incremental costs (£)	Incremental QALYs	ICER (£/QALYs)	Preterm PE cases avoided^a^	£ per preterm PE case avoided
Scenario analysis A—PE incidence (per 10 000 women screened)								
3%^b^	NICE	47761 543	171 858.66	—	—	—	—	—
	FMF^c^	47 758 351	171 859.57	−3191	0.92	Dominant	16.0	−199
5%	NICE	48 943 244	171 856.10	—	—	—	—	—
	FMF^c^	48 746 876	171 857.63	−196 368	1.52	Dominant	26.7	−7364
7%	NICE	50 124 946	171 853.55	—	—	—	—	—
	FMF^c^	49 735 400	171 855.68	−389 545	2.13	Dominant	37.3	−10 435
Scenario analysis B—Aspirin adherence rate (per 10 000 women screened)								
25%	NICE	47 956 220	171 858.13	—	—	—	—	—
	FMF^c^	48 146 356	171 858.43	190 136	0.31	622 547	5.3	35 653
50%	NICE	47 858 881	171 858.39	—	—	—	—	—
	FMF^c^	47 952 354	171 859.00	93 472	0.61	153 024	10.7	8764
75%^b^	NICE	47 761 543	171 858.66	—	—	—	—	—
	FMF^c^	47 758 351	171 859.57	–3191	0.92	Dominant	16.0	−199
100%	NICE	47 664 204	171 858.92	—	—	—	—	—
	FMF^c^	47 564 349	171 860.14	−99 855	1.22	Dominant	21.3	−4681
Scenario analysis C—Different FMF combinations (per 10 000 women screened)								
NICE		47 761 543	171 858.66	—	—	—	—	—
Maternal factors + MAP		47 705 615	171 858.83	−55 927	0.18	Dominant	3.1	−18 114
Maternal factors + MAP + UtA‐PI		47 664 334	171 859.40	−97 209	0.74	Dominant	12.9	−7529
Maternal factors + MAP + PlGF		47 718 222	171 859.27	−43 321	0.61	Dominant	10.7	−4062
Maternal factors + MAP + UtA‐PI + PlGF^b^		47 758 351	171 859.57	−3191	0.92	Dominant	16.0	−199

Abbreviations: FMF, Fetal Medicine Foundation; ICER, incremental cost‐effectiveness ratio; MAP, mean arterial pressure; NICE, National Institute for Health and Care Excellence; PE, preeclampsia; PlGF, placental growth factor; QALY, quality‐adjusted life year; UtA‐PI, uterine artery pulsatility index.

^a^ Additional cases avoided versus the NICE strategy.

^b^ Base‐case analysis.

^c^ FMF strategy using maternal factors + MAP + UtA‐PI + PlGF.

### 3.3 Scenario Analysis B: Aspirin Adherence

Aspirin adherence per 10 000 patients screened was varied at 25%, 50%, 75% (base‐case) and 100% (Table 3). Additional preterm PE cases were avoided with the FMF strategy versus the NICE strategy at 25% and 50% aspirin adherence; however, the FMF strategy was no longer more cost‐effective than NICE as a result of higher costs, despite an increase in QALYs. The FMF strategy was both cost‐saving and associated with higher QALYs versus the NICE strategy at 75% and 100% aspirin adherence, resulting in dominant ICERs. At 100% aspirin adherence, the FMF strategy led to a cost‐saving of £4681 per preterm PE case avoided versus the NICE strategy.

### 3.4 Scenario Analysis C: Biomarker Components of the FMF Strategy

As shown in Table 3, ICERs for all biomarker combinations of the FMF strategy were dominant compared with the NICE strategy. Incremental QALYs for the FMF strategies (versus NICE‐recommended screening) were 0.18 using maternal factors + MAP, 0.74 using maternal factors + MAP + UtA‐PI, and 0.61 using maternal factors + MAP + PlGF. The base‐case FMF strategy (maternal factors + MAP + UtA‐PI + PlGF) demonstrated an incremental QALY gain of 0.92 compared with the NICE strategy and was associated with the greatest number of preterm PE cases avoided and highest incremental QALYs compared with the other biomarker combinations. Therefore, of all biomarker combinations modelled for the FMF strategy, the base‐case FMF strategy displayed the greatest clinical benefit versus NICE‐recommended screening.

### 3.5 Scenario Analyses D–E/Threshold Analyses A‐B

When parameters were modelled across a 1‐year time horizon in scenario analysis D, incremental QALYs for the FMF strategy versus the NICE strategy were 1.31, at an incremental cost‐saving of £3201, resulting in a dominant ICER and a cost‐saving of £200 per preterm PE case avoided (Table S8).

In scenario analysis E, while the FMF strategy remained dominant across 100% hospitalisation rates for both preterm and term PE, at a rate of 80% for preterm and 100% for term PE, the ICER for the FMF strategy versus NICE was £8285/QALY, remaining cost‐effective at NICE WTP thresholds of £20 000–30 000/QALY (Table S9). In threshold analysis A, the FMF strategy remained dominant versus NICE at a preterm PE hospitalisation rate ≥ 91%. At WTP thresholds of £20 000/QALY or £30 000/QALY, the FMF strategy remained cost‐effective across preterm PE hospitalisation rates ≥ 64% and ≥ 50%, respectively (Table S10).

In threshold analysis B, FMF became the dominant strategy at a cost of ≤ £14 for UtA‐PI measurement (Table S11). At WTP thresholds of £20 000/QALY or £30 000/QALY, the FMF strategy remained cost‐effective across UtA‐PI costs of ≤ £15.83 and ≤ £16.70, respectively.

### 3.6 Sensitivity Analyses

The PSA demonstrated that the average total costs estimated from 10 000 replications were £47 763 361 (£4776.33 per patient) for the FMF strategy, and £47 766 199 (£4776.19 per patient) for the NICE strategy, leading to an incremental cost of –£2838 (−£0.28 per patient) for the FMF strategy compared with NICE‐recommended screening (Table S12). Mean total QALYs were 171 899.78 for the FMF strategy and 171 898.86 for the NICE strategy, with an incremental QALY value of 0.92. As with the base‐case analysis, the ICER was dominant for the FMF strategy versus NICE‐recommended screening.

The cost‐effectiveness plane showed that the majority of simulations resulted in lower costs and higher QALYs for the FMF strategy versus NICE (Figure S1); the probability of cost‐effectiveness of the FMF strategy was 59.41% (95% confidence interval [CI]: 58.45–60.37) at a cost‐effectiveness threshold of £20 000/QALY. The narrow 95% CI suggests that the estimated probability was stable and unlikely to change materially with additional simulations. The cost‐effectiveness acceptability curve is shown in Figure S2. The one‐way sensitivity analysis indicated that the most sensitive parameters of those included in the base‐case analysis were sensitivity of the FMF and NICE strategies for predicting preterm PE and proportions of preterm and term PE (Figure S3). EVPI analysis results are reported in Figure S4.

## 4 Discussion

### 4.1 Main Findings

PE represents a significant global health and economic burden, and there remains an unmet need for effective screening regimens in clinical practice in the UK [10, 11, 19]. The FMF ‘triple test’ (maternal factors + MAP + UtA‐PI + PlGF) has shown promising performance for the detection of patients at high‐risk for preterm PE in the first‐trimester, compared with NICE‐recommended screening [6, 9, 21]. As such, the current study evaluated the cost‐effectiveness of the FMF strategy versus the NICE strategy for first‐trimester screening of preterm PE, from the UK NHS and PSS perspective.

This simulation of 10 000 pregnancies demonstrated that the FMF strategy (maternal factors + MAP + UtA‐PI + PlGF) is a cost‐saving and cost‐effective screening method for preterm PE risk in the UK. The FMF strategy was associated with both lower costs and higher QALYs versus NICE‐recommended screening. While all biomarker combinations of the FMF strategy were dominant compared with the NICE strategy, the FMF strategy using maternal factors + MAP + UtA‐PI + PlGF was associated with the greatest clinical efficacy (more preterm PE cases avoided) and higher QALYs versus NICE‐recommended screening. To achieve optimal cost‐effectiveness in the real‐world setting, implementation of the FMF strategy using maternal factors + MAP + UtA‐PI + PlGF would be required. Barriers to implementation (such as the immediate cost of equipment and training, as well as the need for audit and quality control) may impact the potential benefit of the FMF strategy [22].

The FMF strategy was the preferred screening method for preterm PE at plausible levels of PE incidence. This was evidenced by the greater cost‐effectiveness of the FMF strategy, as a result of both an increase in QALYs and reduced costs versus NICE, at 3% (base‐case), 5% and 7% rates of PE incidence. As PE incidence increased, QALYs increased and costs decreased when using the FMF versus NICE strategy. This has real‐world implications as the World Health Organisation reports that PE may occur in up to 8% of pregnancies globally [23].

Treatment with aspirin at a dosage of 150 mg per day from 11 to 13 weeks' until 36 weeks' gestation has been shown to reduce the incidence of preterm PE and preterm birth in high‐risk patients [4, 24]. Furthermore, a meta‐analysis reported that aspirin at a daily dose of ≥ 100 mg administered by 16 weeks' gestation reduces the risk of preterm PE by 67% [25]. Our analysis showed that the FMF strategy was dominant versus NICE‐recommended screening at 75% and 100% aspirin adherence rates. However, variable levels of aspirin adherence in clinical practice may limit the cost‐effectiveness of the FMF strategy. A prior analysis in high‐risk patients (determined by the NICE strategy) reported levels of aspirin adherence from the first trimester to the end of pregnancy as low as 23% [9]. In addition, an aspirin adherence rate of < 90% reduced the incidence of preterm PE by only 40% versus 75% for adherence rates of ≥ 90% [26]. Adherence rates were positively associated with family history of PE and negatively associated with smoking, Afro‐Caribbean and South Asian racial origin, history of PE in a previous pregnancy and maternal age < 25 years [26]. Given variable levels of compliance [9, 26], attention to aspirin adherence will be necessary to retain the lower costs and increased QALYs associated with the FMF strategy.

The FMF strategy was dominant versus the NICE strategy across both the lifetime (base‐case) and 1‐year (scenario analysis) time horizons modelled. While lifetime health consequences were modelled in the base‐case analysis, long‐term cost differences were assumed to be negligible between strategies, as the screening approach modifies the incidence of preterm PE rather than the long‐term disease trajectory once outcomes occur. However, cost implications beyond the perinatal period, including long‐term maternal cardiovascular consequences of PE, require further investigation.

As the probability of hospitalisation parameters was based on expert opinion in the base‐case analysis due to a lack of robust published sources, scenario analysis E modelled various hospitalisation rates to investigate the impact of this parameter on overall outcomes. The FMF strategy remained dominant versus NICE across 100% preterm and term PE hospitalisation rates, and remained cost‐effective at rates of 80% for preterm and 100% for term PE; threshold analysis A indicated that the FMF strategy remained dominant at a preterm PE hospitalisation rate of ≥ 91%. Further research into the probability of hospitalisation for preterm PE is required to reflect real‐world practices and accurately model the cost‐effectiveness of screening approaches.

Threshold analysis B indicated that plausible variations in UtA‐PI costs are unlikely to affect the cost‐effectiveness of the FMF versus NICE strategy. To note, training and equipment costs for UtA‐PI were not modelled as the analysis reflects implementation within existing NHS ultrasound infrastructure; UtA‐PI uses standard Doppler functionality already available in routine NHS scans.

The cost‐effectiveness acceptability curve demonstrated that the FMF strategy had a greater probability of cost‐effectiveness versus the NICE strategy at almost all WTP thresholds, including the NICE‐recommended WTP threshold of £20 000–30 000/QALY. NICE recently announced that this threshold will be raised to £25 000–£35 000/QALY [27]. Notably, the FMF strategy also demonstrated a greater probability of cost‐effectiveness versus the NICE strategy across this WTP threshold.

### 4.2 Strengths and Limitations

The results of the PSA were consistent with the base‐case analysis, indicating a high level of reliability of the model's input parameters. Moreover, the probability of cost‐effectiveness of the FMF strategy was greater than that of the NICE strategy across all measured ICER thresholds, demonstrating the robustness of these findings. These data are supported by previous cost‐effectiveness analyses evaluating different biomarker combinations of the FMF strategy versus NICE‐recommended screening based on maternal factors alone [12, 13, 14, 15]. To our knowledge, this study is the first model‐based cost‐effectiveness analysis of the FMF strategy (maternal factors + MAP + UtA‐PI + PlGF) versus the NICE strategy for first‐trimester screening for preterm PE risk in the UK. Importantly, many input parameters were sourced from Nzelu et al. (2023) [15], which studied a diverse population, thereby supporting the real‐world applicability of our findings.

This study was associated with limitations. Epidemiological and clinical input parameters were based on published literature and expert opinion, which may not fully capture all real‐world variability within the UK. Utilities for some health states were sourced from previously published NICE guidance, and may not perfectly reflect patient‐reported outcomes in current UK practice. Clinical performance data for the FMF strategy were derived primarily from controlled studies and may not fully generalise to all NHS maternity care settings. Costs for UtA‐PI and PlGF testing were assumed to be constant, but may vary by site, implementation model or changes in procurement practices. Similarly, aspirin adherence was applied as a fixed rate per scenario, but real‐world adherence is likely heterogeneous and influenced by demographic, clinical and behavioural factors. Such variations in aspirin adherence in clinical practice may limit cost‐effectiveness of the FMF strategy in routine clinical practice. The model assumed that all patients with PE were treated with labetalol as the first‐line antihypertensive based on NICE recommendations; however, these guidelines recommend nifedipine in patients unsuitable for labetalol [5]; despite treatment variability, as both medications are low cost, labetalol was modelled uniformly to avoid complexity. Additionally, the model accounted for disutility of NICU admission only. However, the inclusion of SCBU disutility would likely further improve the ICER for the FMF strategy versus NICE‐recommended screening as a result of fewer cases of preterm PE and as such, fewer SCBU admissions. The analysis was conducted from the UK NHS and PSS perspective and therefore did not account for indirect societal costs, for example, lost productivity or informal caregiving. Finally, potential psychological impact or downstream health resource use related to receiving a high‐risk or false‐positive screening result was not explicitly captured, as robust evidence quantifying the magnitude and duration of these effects in the context of PE screening is limited. However, a recent study reported minimal psychological impact in patients categorised as high‐risk after PE screening [28]. Moreover, as the specificity of the FMF strategy is slightly greater versus the NICE strategy, potential downstream implications of false‐positive results (e.g., additional monitoring, consultations or psychosocial effects) are unlikely to disproportionately affect the FMF arm or bias the analysis in favour of the intervention.

### 4.3 Interpretation

The cost‐effectiveness of the FMF strategy has been demonstrated outside the UK [12, 13, 14, 15]. In a model of 763 732 patients in Germany, an FMF strategy using maternal factors + MAP + UtA‐PI + PlGF + PAPP‐A, compared with routine clinical risk factor screening, was associated with a decrease in preterm PE at an incremental cost of €3795 per PE case averted [14]. At a WTP threshold of €4200 per case avoided, this combination of FMF biomarker components had a probability of over 50% of being cost‐effective versus routine screening [14]. In model‐based analyses in Switzerland and Belgium, the FMF strategy (maternal factors + MAP + UtA‐PI + PlGF) was dominant compared with SoC screening based on maternal characteristics [13, 14]. Similarly, first‐trimester FMF screening coupled with early low‐dose aspirin resulted in a projected cost‐saving of C$14.39 million for the Canadian healthcare system [12]. Prior to our study, in the UK, cost‐effectiveness analysis of the FMF strategy was limited to a retrospective real‐world analysis [15]. Using a conservative approach, an FMF strategy using maternal factors + MAP + PAPP‐A was associated with seven fewer cases of preterm PE versus the NICE strategy and a cost‐saving of £9.06 per patient screened [15]. Notably, in the current study, the FMF strategy was associated with a cost‐saving of £199 per preterm PE case avoided compared with the NICE strategy and was associated with 16 fewer cases of preterm PE. These data support the inclusion of UtA‐PI and PlGF in the optimal FMF strategy. Furthermore, performance data indicate that inclusion of PAPP‐A (with maternal factors + MAP + UtA‐PI + PlGF) does not significantly improve the performance of FMF screening for preterm PE [6]. As such, the International Society of Ultrasound in Obstetrics and Gynaecology and the International Federation of Gynaecology and Obstetrics recommend the FMF strategy (maternal factors + MAP + UtA‐PI + PlGF) for preterm PE risk screening [8, 29].

## 5 Conclusion

In this simulation model of preterm PE risk screening in patients at 11–13 weeks' gestation in the UK, the FMF strategy (maternal factors + MAP + UtA‐PI + PlGF) demonstrated increased cost‐effectiveness compared with NICE‐recommended clinical risk factor scoring. Cost‐effectiveness of the FMF strategy was dependent on achieving high aspirin adherence and should be interpreted in light of modelling assumptions. These findings contribute to the evidence base informing discussions around implementation of updated national recommendations in the UK.

## Author Contributions

All authors provided substantial contributions to conception and design, or acquisition of data, or analysis and interpretation of data; and drafting the article or revising it critically for important intellectual content; and final approval of the version to be published and agreement to be accountable for all aspects of the work in ensuring that questions related to the accuracy or integrity of any part of the work are appropriately investigated and resolved.

## Funding

This analysis was funded by Roche Diagnostics UK and Ireland. The model structure and key assumptions were based on published literature, expert clinical input and publicly available NHS reference costs where available, as opposed to proprietary sources. The academic and clinical co‐authors were actively involved in the study design, interpretation of the results and review and approval of the final manuscript, ensuring that the analysis and conclusions reflect multidisciplinary clinical and methodological input.

## Ethics Statement

The authors have nothing to report.

## Consent

The authors have nothing to report.

## Conflicts of Interest

L.C.P. received speaker honoraria from Mindray Medical, Sonoscape, Samsung Healthcare, GE HealthCare and Thermo Fisher Scientific and received in‐kind contributions from astraia, Revvity, Thermo Fisher Scientific, Roche Diagnostics, Ningbo Aucheer and GE HealthCare. M.A.A. is an employee of Roche Diagnostics Ltd. A.J.A. is an employee of Roche Diagnostics Ltd. and holds shares in Roche. The other authors declare no conflicts of interest.

## Supporting information


**Table S1:** Probability parameters.
**Table S2:** Cost parameters.
**Table S3:** Utility and disutility parameters.^a^
**Table S4:** Length of hospital stay parameters.
**Table S5:** Scenario analysis parameters.
**Table S6:** Base‐case clinical outcomes (per 10 000 patients screened).
**Table S7:** Base‐case costs breakdown (per one patient screened).
**Table S8:** Scenario analysis D: 1‐year time horizon.
**Table S9:** Scenario analysis E: Probability of hospitalisation.
**Table S10:** Threshold analysis A: Probability of hospitalisation.
**Table S11:** Threshold analysis B: Cost of UtA‐PI.
**Table S12:** Probabilistic sensitivity analysis (per 10 000 patients screened).
**Figure S1:** Scatter plot of the probabilistic sensitivity analysis.
**Figure S2:** Cost‐effectiveness acceptability curve.
**Figure S3:** One‐way sensitivity analysis tornado diagram.^a^
**Figure S4:** Expected value of perfect information analysis.

## Data Availability

The data that support the findings of this study are available from the corresponding author upon reasonable request.
