# Supplementary material for: First Trimester Screening for Preterm Preeclampsia in the United Kingdom: A Cost‐Effectiveness Analysis
Source: BJOG. 2026 Jul 1;133(12):2178–87. doi: 10.1111/1471-0528.70294 (PMC13659417; doi:10.1111/1471-0528.70294)
Supplement: Supplementary file 1 [file BJO-133-2178-s001.docx]

# Supplementary material

## Table S1. Probability parameters

| **Parameter** | **Estimate** | **Distribution** | **Alpha** | **Beta** | **95% CI** | **Reference** |
| --- | --- | --- | --- | --- | --- | --- |
| Hospitalisation preterm PE | 96.00% | Beta | 96 | 4 | 0.91–0.99 | Expert opinion |
| Hospitalisation term PE | 96.00% | Beta | 96 | 4 | 0.91–0.99 | Expert opinion |
| NICU preterm PE | 41.00% | Beta | 59 | 85 | 0.33–0.49 | Nzelu et al, 2023^1,a^ |
| NICU term PE | 9.40% | Beta | 91 | 873 | 0.077–0.12 | Nzelu et al, 2023^1,a^ |
| NICU no PE | 5.90% | Beta | 94 | 1501 | 0.048–0.071 | Nzelu et al, 2023^1,a^ |
| SCBU preterm PE | 12.00% | Beta | 88 | 645 | 0.098–0.14 | Nzelu et al, 2023^1,a^ |
| SCBU term PE | 2.60% | Beta | 97 | 3649 | 0.021–0.031 | Nzelu et al, 2023^1,a^ |
| SCBU no PE | 1.40% | Beta | 99 | 6944 | 0.011–0.017 | Nzelu et al, 2023^1,a^ |
| Stillbirth preterm PE | 1.53% | Beta | 77 | 4942 | 0.012–0.019 | Harmon et al, 2015^2,b^ |
| Stillbirth term PE | 0.20% | Beta | 32 | 15969 | 0.0014–0.0027 | Harmon et al, 2015^2,b^ |
| Stillbirth no PE | 0.30% | Beta | 1838 | 533137 | 0.003–0.0036 | Harmon et al, 2015^2,b^ |
| Livebirth preterm PE | 98.47% | - | - | - | 0.981–0.988 | Harmon et al, 2015^2,b,c^ |
| Livebirth term PE | 99.80% | - | - | - | 0.997–0.999 | Harmon et al, 2015^2,b,c^ |
| Livebirth no PE | 99.70% | - | - | - | 0.996–0.997 | Harmon et al, 2015^2,b,c^ |
| Maternal death preterm PE | 0.03% | Beta | 100 | 333132 | 0.00024–0.00036 | Assumption based on unpublished Roche Global Model |
| Maternal death term PE | 0.03% | Beta | 100 | 333132 | 0.00024–0.00036 | Assumption based on unpublished Roche Global Model |
| Maternal death no PE | 0.02% | Beta | 100 | 499799 | 0.00016–0.00024 | Assumption based on unpublished Roche Global Model |
| Maternal survival preterm PE | 99.97% | - | - | - | 0.9996–0.9998 | Assumption based on unpublished Roche Global Model^d^ |
| Maternal survival term PE | 99.97% | - | - | - | 0.9996–0.9998 | Assumption based on unpublished Roche Global Model^d^ |
| Maternal survival no PE | 99.98% | - | - | - | 0.9997–0.9998 | Assumption based on unpublished Roche Global Model^d^ |
| Vaginal delivery preterm PE | 20.40% | Beta | 80 | 311 | 0.17–0.25 | Nzelu et al, 2023^1,a^ |
| Vaginal delivery term PE | 46.10% | Beta | 54 | 63 | 0.37–0.55 | Nzelu et al, 2023^1,a^ |
| Vaginal delivery no PE | 61.70% | Beta | 38 | 24 | 0.49–0.73 | Nzelu et al, 2023^1,a^ |
| Elective caesarean delivery preterm PE | 8.20% | Beta | 92 | 1028 | 0.067–0.099 | Nzelu et al, 2023^1,a^ |
| Elective caesarean delivery term PE | 10.90% | Beta | 89 | 728 | 0.089–0.13 | Nzelu et al, 2023^1,a^ |
| Elective caesarean delivery no PE | 17.10% | Beta | 83 | 402 | 0.14–0.21 | Nzelu et al, 2023^1,a^ |
| Emergency caesarean delivery preterm PE | 71.40% | Beta | 29 | 11 | 0.58–0.85 | Nzelu et al, 2023^1,a^ |
| Emergency caesarean delivery term PE | 43.00% | Beta | 57 | 76 | 0.35–0.51 | Nzelu et al, 2023^1,a^ |
| Emergency caesarean delivery no PE | 21.20% | Beta | 79 | 293 | 0.17–0.26 | Nzelu et al, 2023^1,a^ |

^a^Estimates were taken directly from cited source; ^b^Estimates were calculated from cited source; ^c^Estimates were not individually sampled and were calculated based on 1-stillbirth to ensure livebirth and stillbirth estimates add up to 1 in each of the 10,000 replications to avoid inconsistencies; ^d^Estimates were not individually sampled and were calculated based on 1-maternal death to ensure maternal survival and maternal death estimates add up to 1 in each of the 10,000 replications to avoid inconsistencies.
CI, confidence interval; NICU, neonatal intensive care unit; PE, preeclampsia; SCBU, specialty care baby unit.

## Table S2. Cost parameters

| **Parameter** | **Estimate (£)** | **Distribution** | **Alpha** | **Beta** | **95% CI** | **Reference** |
| --- | --- | --- | --- | --- | --- | --- |
| Maternal factors | 0 | - | - | - | - | Expert opinion |
| MAP | 0 | - | - | - | - | Expert opinion |
| UtA-PI | 13.68 | Gamma | 100 | 0.14 | 11.39–16.87 | Drouin et al, 2017^3,a^/NHS reference costs^4,b^ |
| PlGF | 15.00 | Gamma | 100 | 0.15 | 12–18 | Internal Roche price assumption^c^ |
| Vaginal delivery preterm PE | 3137.00 | Gamma | 100 | 31.37 | 2552–3781 | NHS reference costs^4,b^ |
| Vaginal delivery term PE | 2299.00 | Gamma | 100 | 22.99 | 1871–2771 | NHS reference costs^4,b^ |
| Vaginal delivery no PE | 2285.00 | Gamma | 100 | 22.85 | 1859–2754 | NHS reference costs^4,b^ |
| Elective caesarean delivery preterm PE | 6357.00 | Gamma | 100 | 63.57 | 5172–7662 | NHS reference costs^4,b^ |
| Elective caesarean delivery term PE | 5292.00 | Gamma | 100 | 52.92 | 4306–6378 | NHS reference costs^4,b^ |
| Elective caesarean delivery no PE | 4652.00 | Gamma | 100 | 46.52 | 3785–5607 | NHS reference costs^4,b^ |
| Emergency caesarean delivery preterm PE | 9564.00 | Gamma | 100 | 95.64 | 7782–11527 | NHS reference costs^4,b^ |
| Emergency caesarean delivery term PE | 7751.00 | Gamma | 100 | 77.51 | 6307–9342 | NHS reference costs^4,b^ |
| Emergency caesarean delivery no PE | 6604.00 | Gamma | 100 | 66.04 | 5373–7960 | NHS reference costs^4,b^ |
| Hospitalisation preterm PE | 4212.00 | Gamma | 100 | 42.12 | 3427–5077 | NHS reference costs^4,b^ |
| Hospitalisation term PE | 923.00 | Gamma | 100 | 9.23 | 751–1112 | NHS reference costs^4,b^ |
| Aspirin | 10.75 | Gamma | 100 | 0.11 | 8.95–13.26 | BNF^5,d^ |
| Corticosteroids | 9.85 | Gamma | 100 | 0.10 | 8.14–12.05 | BNF^6,e^ |
| Labetalol | 8.55 | Gamma | 100 | 0.09 | 7.32–10.85 | BNF^7,f^ |
| First midwife visit | 247.00 | Gamma | 100 | 2.47 | 201–298 | NHS reference costs^4,b^ |
| Follow-up midwife visit | 126.00 | Gamma | 100 | 1.26 | 103–152 | NHS reference costs^4,b^ |
| Blood pressure | 0.00 | - | - | - | - | Expert opinion |
| Kidney function test | 2.00 | Gamma | 100 | 0.02 | 1.63–2.41 | NHS reference costs^4,b^ |
| Fetal assessment | 165.00 | Gamma | 100 | 1.65 | 134–199 | NHS reference costs^4,b^ |
| Blood tests | 5.00 | Gamma | 100 | 0.05 | 4.07–6.03 | NHS reference costs^4,b^ |
| Maternal death | 0.00 | - | - | - | - | Assumption |
| Stillbirth | 2458.00 | Gamma | 100 | 24.58 | 2000–2963 | Campbell et al, 2018^8,g^ |
| NICU | 1861.00 | Gamma | 100 | 18.61 | 1514–2243 | NHS reference costs^9,h^ |
| SCBU | 957.00 | Gamma | 100 | 9.57 | 779–1153 | NHS reference costs^9,h^ |

^a^Cost for UtA-PI was modelled as an incremental addition to the first trimester ultrasound. This cost was assumed based on the unit cost per minute for the routine ultrasound scan using the NHS reference cost of £171 (adjusted for inflation to 2024),^4^ assuming a length of 25 minutes based on expert opinion, resulting in a cost of £6.84 per minute. A conservative estimate of an additional two minutes to obtain measurements was applied, based on 106–118 seconds reported by Drouin et al, 2017.^3^ ^b^Estimates sourced from 2022 NHS reference costs and adjusted for inflation to 2024; ^c^Estimated average cost to NHS for PlGF as part of FMF screening overall; ^d^Cost calculated for 150 mg taken daily for 175 days; ^e^Cost calculated for four ampules; ^f^Cost calculated for 200 mg twice daily for 28 days; ^g^Estimate taken from source and adjusted for inflation to 2024; ^h^Estimates were taken directly from cited source.
BNF, British National Formulary; CI, confidence interval; MAP, mean arterial pressure; MF, maternal factors; NHS, National Health Service; NICU, neonatal intensive care unit; PE, preeclampsia; PlGF, placental growth factor; SCBU, specialty care baby unit; UtA-PI, uterine artery pulsatility index.

## Table S3. Utility and disutility^a^ parameters

| **Parameter** | **Estimate** | **Distribution** | **Alpha** | **Beta** | **95% CI** | **Reference** |
| --- | --- | --- | --- | --- | --- | --- |
| Aspirin | −0.0002 | Beta | 99.98 | 499800.00 | –0.00024 to  –0.00016 | Nzelu et al, 2023^1^ |
| Maternal death | 0.0000 | - | - | - | - | Assumption |
| Alive mother whose child had no/minor complications | 17.4200 | Normal | 17.42 | 1.74 | 14–20.83 | NICE HTG630^10^ |
| Alive mother with stillbirth | 13.4500 | Normal | 13.45 | 1.35 | 10.80–16.10 | NICE HTG630^10^ |
| Vaginal delivery | −0.0079 | Beta | 99.21 | 12523.43 | –0.0094 to  –0.0064 | NICE HTG630^10,b^ |
| Elective caesarean delivery | −0.0337 | Beta | 96.63 | 2770.73 | –0.041 to  –0.027 | NICE HTG630^10,b^ |
| Emergency caesarean delivery | −0.0298 | Beta | 97.02 | 3158.11 | –0.036 to  –0.024 | NICE HTG630^10,b^ |
| 3–6 weeks post-partum | −0.1498 | Beta | 85.03 | 482.75 | –0.18 to  –0.12 | NICE HTG630^10,b^ |
| 12−24 weeks post-partum | −0.1998 | Beta | 80.02 | 320.41 | –0.24 to  –0.16 | NICE HTG630^10,b^ |
| NICU baby (daily) | −0.0001 | Beta | 99.99 | 935700.21 | –0.00013 to  –0.000087 | NICE HTG630^10,b^ |

^a^Disutilities were sampled from beta distributions bounded between 0 and 1, after which the sampled values were multiplied by –1 before being applied to the model, allowing them to represent utility decrements. These disutilities were then applied additively to the baseline utility values for the relevant health states.

CI, confidence interval; NICE, National Institute for Health and Care Excellence; NICU, neonatal intensive care unit.

## Table S4. Length of hospital stay parameters

| **Parameter** | **Estimate (days)** | **Distribution** | **Alpha** | **Beta** | **95% CI** | **Reference** |
| --- | --- | --- | --- | --- | --- | --- |
| NICU preterm PE | 13 | Gamma | 100 | 0.13 | 10.58–15.67 | Nzelu et al, 2023^1^ |
| NICU term PE | 3 | Gamma | 100 | 0.03 | 2.44–3.62 | Nzelu et al, 2023^1^ |
| NICU no PE | 4 | Gamma | 100 | 0.04 | 3.25–4.82 | Nzelu et al, 2023^1^ |
| SCBU preterm PE | 3 | Gamma | 100 | 0.03 | 2.44–3.62 | Nzelu et al, 2023^1^ |
| SCBU term PE | 3 | Gamma | 100 | 0.03 | 2.44–3.62 | Nzelu et al, 2023^1^ |
| SCBU no PE | 13 | Gamma | 100 | 0.03 | 10.58–15.67 | Nzelu et al, 2023^1^ |

CI, confidence interval; NICU, neonatal intensive care unit; PE, preeclampsia; SCBU, specialty care baby unit.

## Table S5. Scenario analysis parameters

| **Parameter** | **Estimate** | **Distribution** | **Alpha** | **Beta** | **95% CI** | **Reference** |
| --- | --- | --- | --- | --- | --- | --- |
| **Sensitivity preterm PE** | | | | | | |
| NICE | 41.5% | Beta | 59 | 83 | 0.34–0.50 | Tan et al, 2018^11,a^ |
| Maternal factors+MAP | 49.3% | Beta | 70 | 72 | 0.41–0.57 | Tan et al, 2018^11,a^ |
| Maternal factors+MAP+ UtA-PI | 73.9% | Beta | 105 | 37 | 0.66–0.81 | Tan et al, 2018^11,a^ |
| Maternal factors+MAP+ PlGF | 68.3% | Beta | 97 | 45 | 0.60–0.76 | Tan et al, 2018^11,a^ |
| Maternal factors+MAP+ UtA-PI+PlGF | 81.7% | Beta | 116 | 26 | 0.75–0.88 | Tan et al, 2018^11,a^ |
| **Specificity preterm PE** | | | | | | |
| NICE | 90.1% | Beta | 14658 | 1616 | 0.90–0.91 | Tan et al, 2018^11,a^ |
| Maternal factors+MAP | 90.1% | Beta | 14669 | 1605 | 0.90–0.91 | Tan et al, 2018^11,a^ |
| Maternal factors+MAP+ UtA-PI | 90.4% | Beta | 14704 | 1570 | 0.90–0.91 | Tan et al, 2018^11,a^ |
| Maternal factors+MAP+ PlGF | 90.3% | Beta | 14696 | 1578 | 0.90–0.91 | Tan et al, 2018^11,a^ |
| Maternal factors+MAP+ UtA-PI+PlGF | 90.4% | Beta | 14715 | 1559 | 0.90–0.91 | Tan et al, 2018^11,a^ |
| **Sensitivity term PE** | | | | | | |
| NICE | 30.2% | Beta | 100 | 231 | 0.25–0.35 | Tan et al, 2018^11,a^ |
| Maternal factors+MAP | 38.7% | Beta | 128 | 203 | 0.34–0.44 | Tan et al, 2018^11,a^ |
| Maternal factors+MAP+ UtA-PI | 43.5% | Beta | 144 | 187 | 0.38–0.49 | Tan et al, 2018^11,a^ |
| Maternal factors+MAP+ PlGF | 39.6% | Beta | 131 | 200 | 0.34–0.45 | Tan et al, 2018^11,a^ |
| Maternal factors+MAP+ UtA-PI+PlGF | 42.6% | Beta | 141 | 190 | 0.37–0.48 | Tan et al, 2018^11,a^ |
| **Specificity term PE** | | | | | | |
| NICE | 90.3% | Beta | 14699 | 1575 | 0.90–0.91 | Tan et al, 2018^11,a^ |
| Maternal factors+MAP | 90.5% | Beta | 14727 | 1547 | 0.90–0.91 | Tan et al, 2018^11,a^ |
| Maternal factors+MAP+ UtA-PI | 90.6% | Beta | 14743 | 1531 | 0.90–0.91 | Tan et al, 2018^11,a^ |
| Maternal factors+MAP+ PlGF | 90.5% | Beta | 14730 | 1544 | 0.90–0.91 | Tan et al, 2018^11,a^ |
| Maternal factors+MAP+ UtA-PI+PlGF | 90.6% | Beta | 14740 | 1534 | 0.90–0.91 | Tan et al, 2018^11,a^ |
| **Epidemiology** | | | | | | |
| Incidence 1 | 3% | Beta | 97 | 3136 | 0.024–0.036 | Expert opinion |
| Incidence 2 | 5% | - | - | - | - | Expert opinion |
| Incidence 3 | 7% | - | - | - | - | Expert opinion |
| **Aspirin adherence** | | | | | | |
| Rate 1 | 100% |  | - | - |  | Expert opinion |
| Rate 2 | 75% |  | - | - |  | Expert opinion |
| Rate 3 | 50% |  | - | - |  | Expert opinion |
| Rate 4 | 25% |  | - | - |  | Expert opinion |
| **One-year time horizon** | | | | | | |
| Starting mother utility | 0.911 | - | - | - | - | Nzelu et al, 2023^1^ |
| Vaginal delivery (daily) | –0.0079 | - | - | - | - | Nzelu et al, 2023^1,b^ |
| Caesarean delivery (daily) | –0.033 | - | - | - | - | Nzelu et al, 2023^1,b^ |
| Develops PE | –0.03 | - | - | - | - | Nzelu et al, 2023^1^ |
| Stillbirth | –0.08 | - | - | - | - | Nzelu et al, 2023^1^ |
| NICU admission (daily) | –0.008 | - | - | - | - | Nzelu et al, 2023^1^ |
| Aspirin | −0.0002 | - | - | - | - | Nzelu et al, 2023^1^ |

^a^Values were calculated from the cited manuscript; ^b^Values from the cited manuscript were extrapolated to the one-year time horizon.
CI, confidence interval; MAP, mean arterial pressure; NICE, National Institute for Health and Care Excellence; PE, preeclampsia; PlGF, placental growth factor; UtA-PI, uterine artery pulsatility index.

## Table S6. Base-case clinical outcomes (per 10,000 patients screened)

| **Cases** | **NICE** | **FMF** | **Difference** | **% reduction** |
| --- | --- | --- | --- | --- |
| Preterm PE | 69.15 | 53.15 | −16.0 | 23 |
| Term PE | 214.29 | 214.29 | 0.0 | 0 |
| All PE | 283.44 | 267.44 | −16.0 | 6 |
| Stillbirths | 34.98 | 34.79 | −0.19 | 1 |
| Maternal deaths | 2.0283 | 2.0267 | −0.0016 | 0.1% |

FMF, Fetal Medicine Foundation; NICE, National Institute for Health and Care Excellence; PE, preeclampsia.

## Table S7. Base-case costs breakdown (per one patient screened)

| **Costs** | **NICE (£)** | **FMF (£)** | **Difference (£)** |
| --- | --- | --- | --- |
| Screening | 0.00 | 29.00 | 29.00 |
| Midwife visits | 373.00 | 373.00 | 0.00 |
| NICU | 504.04 | 489.12 | −15.00 |
| SCBU | 172.60 | 172.33 | −0.27 |
| Delivery | 3,664.94 | 3,657.92 | −7.01 |
| Aspirin | 0.84 | 0.87 | 0.03 |
| Hospitalisation | 46.95 | 40.48 | −6.47 |
| Treatment | 0.31 | 0.28 | −0.03 |
| Management | 4.88 | 4.60 | −0.28 |
| Total | 4,776.15 | 4,775.84 | −0.32 |

FMF, Fetal Medicine Foundation; NICE, National Institute for Health and Care Excellence; NICU, neonatal intensive care unit; SCBU, specialty care baby unit.

## Table S8. Scenario analysis D: One-year time horizon

| **Strategy** | **Total costs (£)** | **Total QALYs** | **Incremental costs (£)** | **Incremental QALYs** | **ICER (£/QALY)** | **Cost per preterm PE case avoided (£)** |
| --- | --- | --- | --- | --- | --- | --- |
| **NICE** | 47,761,553 | 8,897.01 | - | - | - | - |
| **FMF (maternal factors+ MAP+ UtA-PI +PlGF)** | 47,758,351 | 8,898.32 | –3,201 | 1.31 | Dominant | –200 |

FMF, Fetal Medicine Foundation; ICER, incremental cost-effectiveness ratio; MAP, mean arterial pressure; NICE, National Institute for Health and Care Excellence; PE, preeclampsia; PlGF, placental growth factor; QALY, quality-adjusted life year; UtA-PI, uterine artery pulsatility index.

## Table S9. Scenario analysis E: Probability of hospitalisation

| **Strategy** | **Total costs (£)** | **Total QALYs** | **Incremental costs (£)** | **Incremental QALYs** | **ICER (£/QALY)** | **Cost per preterm PE Case avoided** |
| --- | --- | --- | --- | --- | --- | --- |
| **100% preterm PE/100% term PE** | | | | | | |
| **NICE** | 47,781,105 | 171,585.66 | - | - | - | - |
| **FMF (maternal factors+ MAP+ UtA-PI+ PlGF** | 47,775,218 | 171,859.57 | –5,887 | 0.92 | Dominant | –368 |
| **80% preterm PE/100% term PE** | | | | | | |
| **NICE** | 47,722,850 | 171,858.66 | - | - | - | - |
| **FMF (maternal factors+ MAP+ UtA-PI+ PlGF** | 47,730,441 | 171,859.57 | 7,591 | 0.92 | 8,285 | 474 |

FMF, Fetal Medicine Foundation; ICER, incremental cost-effectiveness ratio; MAP, mean arterial pressure; NICE, National Institute for Health and Care Excellence; PE, preeclampsia; PlGF, placental growth factor; QALY, quality-adjusted life year; UtA-PI, uterine artery pulsatility index.

## Table S10. Threshold analysis A: Probability of hospitalisation

| **Cost-effectiveness threshold** | **Rate of hospitalisation (preterm PE)** |
| --- | --- |
| £0/QALY | 91% |
| £10,000/QALY | 78% |
| £20.000/QALY | 64% |
| £30,000/QALY | 50% |

PE, preeclampsia; QALY, quality-adjusted life year.

## Table S11. Threshold analysis B: Cost of UtA-PI

| **Cost-effectiveness threshold** | **Cost of UtA-PI (£)** |
| --- | --- |
| £0/QALY | 14.00 |
| £10,000/QALY | 14.92 |
| £20,000/QALY | 15.83 |
| £30,000/QALY | 16.70 |

QALY, quality-adjusted life year; UtA-PI, uterine artery pulsatility index.

## Table S12. Probabilistic sensitivity analysis (per 10,000 patients screened)

| **Strategy** | **Total costs, £** | **Total QALYs** | **Incremental costs, £  (95% CI)** | **Incremental QALYs  (95% CI)** | **ICER, £/QALY** | **Preterm PE cases avoided (95% CI)** | **% CE  (95% CI)** |
| --- | --- | --- | --- | --- | --- | --- | --- |
| NICE | 47,766,199 | 171,898.86 | - | - | - | - | - |
| FMF (maternal factors+MAP+UtA-PI+PlGF) | 47,763,361 | 171,899.78 | −2,837.71 (−146,142.85 to 115,761.74) | 0.92  (0.091–1.97) | Dominant | 16.0 (10.6−22.3) | 59.41%  (58.45–60.37) |

CE, cost-effectiveness; CI, confidence interval; FMF, Fetal Medicine Foundation; ICER, incremental cost-effectiveness ratio; MAP, mean arterial pressure; NICE, National Institute for Health and Care Excellence; PlGF, placental growth factor; QALY, quality-adjusted life year; UtA-PI, uterine artery pulsatility index.

## Figure S1. Scatter plot of the probabilistic sensitivity analysis

QALY, quality-adjusted life year.

## Figure S2. Cost-effectiveness acceptability curve

FMF, Fetal Medicine Foundation; ICER, incremental cost-effectiveness ratio; NICE, National Institute for Health and Care Excellence; QALY, quality-adjusted life year; WTP, willingness-to-pay.

## Figure S3. One-way sensitivity analysis tornado diagram^a^

^a^Only the top 15 parameters with the greatest impact on cost-effectiveness when varied are included in the plot.

FMF, Fetal Medicine Foundation; LOS, length of stay; NICE, National Institute for Health and Care Excellence; NICU, neonatal intensive care unit; PE, preeclampsia; PlGF, placental growth factor; UtA-PI, uterine artery pulsatility index.

## Figure S4. Expected value of perfect information analysis

EVPI, expected value of perfect information; ICER, incremental cost-effectiveness ratio; QALY, quality-adjusted life year; WTP, willingness-to-pay.

# References

1.	Nzelu D, Palmer T, Stott D, et al. First trimester screening for pre-eclampsia and targeted aspirin prophylaxis: a cost-effectiveness cohort study. BJOG: An International Journal of Obstetrics & Gynaecology. 2024;131(2):222-30.

2.	Harmon QE, Huang L, Umbach DM, et al. Risk of fetal death with preeclampsia. Obstet Gynecol. 2015;125(3):628-35.

3.	Drouin O, Johnson JA, Chaemsaithong P, et al. Transverse technique: complementary approach to measurement of first-trimester uterine artery Doppler. Ultrasound Obstet Gynecol. 2018;52(5):639-47.

4.	NHS England. NHS England » National Cost Collection for the NHS 2022 [Available from: <https://www.england.nhs.uk/publication/2022-23-national-cost-collection-data-publication-2/>.

5.	National Institute for Health and Care Excellence. British National Formulary | Aspirin: @NICEcomms; 2025 [Available from: <https://bnf.nice.org.uk/drugs/aspirin/medicinal-forms/#gastro-resistant-tablet>.

6.	National Institute for Health and Care Excellence. British National Formulary | Dexamethasone: @NICEcomms; 2025 [Available from: <https://bnf.nice.org.uk/drugs/dexamethasone/medicinal-forms/#solution-for-injection>.

7.	National Institute for Health and Care Excellence. British National Formulary | Labetalol hydrochloride: @NICEcomms; 2025 [Available from: <https://bnf.nice.org.uk/drugs/labetalol-hydrochloride/medicinal-forms/#oral-tablet>.

8.	Campbell HE, Kurinczuk JJ, Heazell A, et al. Healthcare and wider societal implications of stillbirth: a population-based cost-of-illness study. Bjog. 2018;125(2):108-17.

9.	NHS England. NHS England » National Cost Collection for the NHS 2024 [Available from: <https://www.england.nhs.uk/publication/2024-25-national-cost-collection-data-publication/>.

10.	National Institute for Health and Care Excellence. PLGF-based testing to help diagnose suspected preterm pre-eclampsia | Guidance | NICE | Committee papers 2022 [Available from: <https://www.nice.org.uk/guidance/htg630/documents/committee-papers>.

11.	Tan MY, Wright D, Syngelaki A, et al. Comparison of diagnostic accuracy of early screening for pre-eclampsia by NICE guidelines and a method combining maternal factors and biomarkers: results of SPREE. Ultrasound in Obstetrics & Gynecology. 2018;51(6):743-50.
